# Effects of cochlear hair cell ablation on spatial learning/memory

**DOI:** 10.1038/s41598-020-77803-7

**Published:** 2020-11-26

**Authors:** Z. Jason Qian, Anthony J. Ricci

**Affiliations:** Department of Otolaryngology-Head and Neck Surgery, Stanford University School of Medicine, 240 Pasteur Drive, Biomedical Innovations Building, R0551, Palo Alto, CA 94304 USA

**Keywords:** Auditory system, Cognitive neuroscience

## Abstract

Current clinical interest lies in the relationship between hearing loss and cognitive impairment. Previous work demonstrated that noise exposure, a common cause of sensorineural hearing loss (SNHL), leads to cognitive impairments in mice. However, in noise-induced models, it is difficult to distinguish the effects of noise trauma from subsequent SNHL on central processes. Here, we use cochlear hair cell ablation to isolate the effects of SNHL. Cochlear hair cells were conditionally and selectively ablated in mature, transgenic mice where the human diphtheria toxin (DT) receptor was expressed behind the hair-cell specific *Pou4f3* promoter. Due to higher *Pou4f3* expression in cochlear hair cells than vestibular hair cells, administration of a low dose of DT caused profound SNHL without vestibular dysfunction and had no effect on wild-type (WT) littermates. Spatial learning/memory was assayed using an automated radial 8-arm maze (RAM), where mice were trained to find food rewards over a 14-day period. The number of working memory errors (WME) and reference memory errors (RME) per training day were recorded. All animals were injected with DT during P30–60 and underwent the RAM assay during P90–120. SNHL animals committed more WME and RME than WT animals, demonstrating that isolated SNHL affected cognitive function. Duration of SNHL (60 versus 90 days post DT injection) had no effect on RAM performance. However, younger age of acquired SNHL (DT on P30 versus P60) was associated with fewer WME. This describes the previously undocumented effect of isolated SNHL on cognitive processes that do not directly rely on auditory sensory input.

## Introduction

Auditory sensory loss is associated with cognitive decline^1,2^. This association is commonly seen in the elderly, who experience a wide clinical spectrum of hearing and cognitive impairments. While this association is likely modulated in part by common aging processes that affect both hearing and cognition^3,4^, current clinical interest lies in whether peripheral hearing loss can induce or accelerate cognitive decline. The cause-and-effect relationship between hearing loss and cognitive decline is plausible due to the dynamic interaction between auditory and cognitive processing. Sensory information processing is a fundamental component of learning, memory, and cognition^5,6^, and brain structures responsible for encoding spatial representations can use visual, olfactory, and auditory information to generate memories of navigable space^7–9^. Progressive hearing loss is correlated with widespread changes in neurotransmitter expression that potentiate spatial memory impairments^1,10,11^. These associations suggest there exists a causal mechanism underlying hearing-related cognitive decline. Determining the causal mechanism would be important to inform whether cognitive decline could be prevented or managed by early auditory interventions.

To begin understanding the causal mechanisms by which hearing loss and cognitive decline are linked, it is important to study how the brain functionally adjusts to the initial loss of auditory input. Noise-induced hearing loss is a common form of acquired hearing loss and is easily induced experimentally. Animals with hearing loss following noise exposure experience decreased hippocampal cell proliferation and synapse formation that correlate with spatial memory impairment^12–14^. However, noise exposure also directly causes cognitive deficits by oxidative stress along both the peripheral and central auditory pathways^15,16^. Therefore, distinguishing the indirect cognitive effects of the subsequent hearing loss from the direct effects of oxidative stress is difficult in the noise induced model.

Here, a transgenic mouse line where hearing loss can be induced through selective cochlear hair cell ablation is used to assess the initial cognitive consequences of hearing loss. We show here that sudden loss of function at the auditory periphery causes a spatial learning and memory impairment. The severity of the impairment persists beyond 2 months of the auditory insult. Mice that lost hearing at an older age (2 months) demonstrated a more severe spatial memory deficit than mice that lost hearing at a younger age (1 month), suggesting that cognitive impairment due to hearing loss may be more severe with advanced age of hearing loss onset. Taken together, this data indicates that sudden hearing loss is accompanied by spatial learning and memory impairment, demonstrating that hearing loss is causally linked to cognitive impairment.

## Results

### Auditory damage

**Figure 1:**
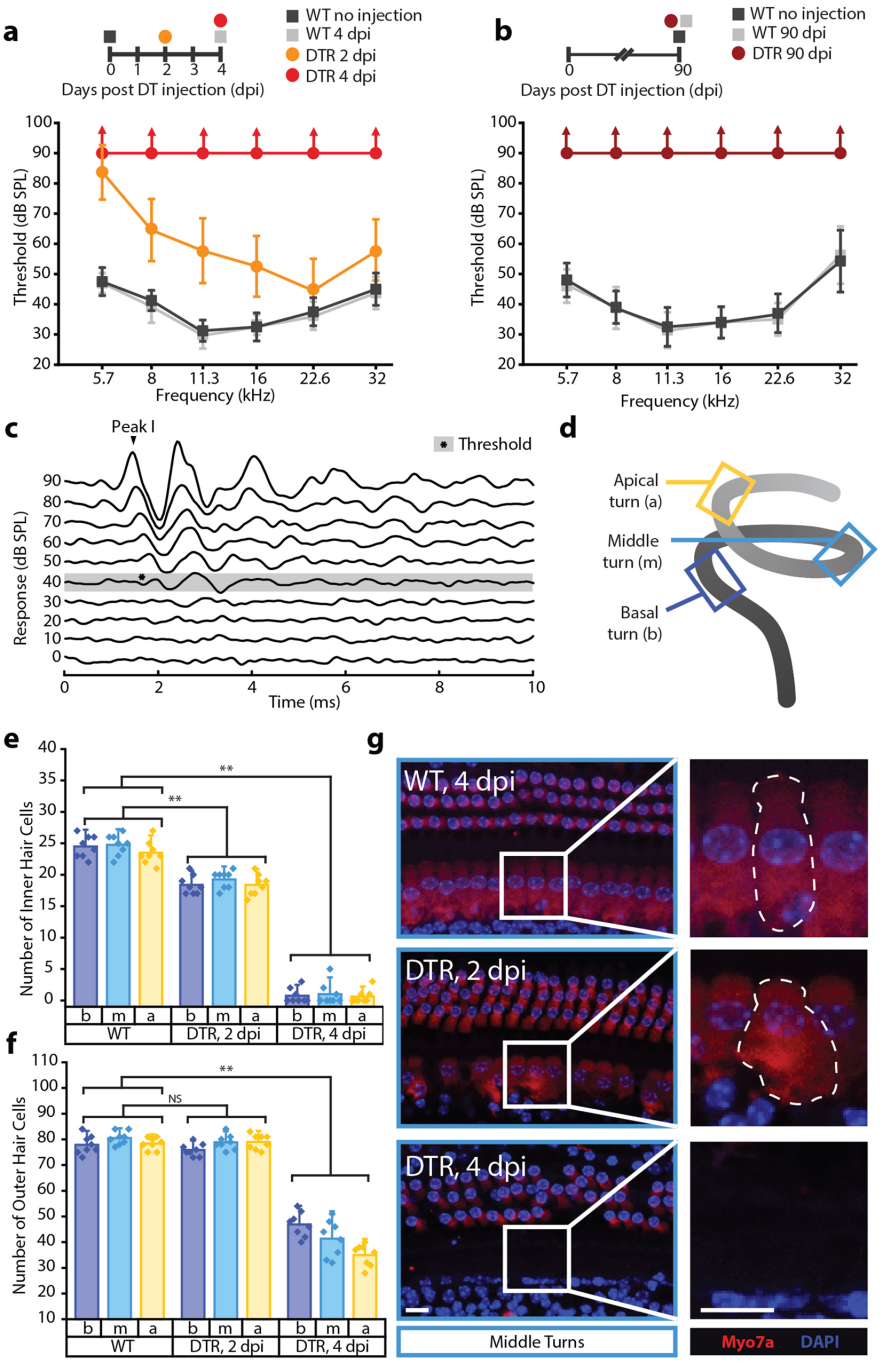
Characterization of cochlear damage. Error bars represent standard deviation, *P < 0.05, **P < 0.001. All scale bars represent 5 μm. (**A**) ABR thresholds from 0–4 days post injection (dpi) show that profound hearing loss occurs by 4 dpi in mature animals (0 dpi = P30, n = 8 ears from 4 mice per group). (**B**) ABR thresholds at 90 dpi show that injection of WT animals have no long term effects on hearing while profound hearing loss is sustained in DTR animals (0 dpi = P30, n = 8 ears from 4 mice per group). (**C**) Auditory thresholds were determined to be lowest intensity at which a characteristic Peak I could be identified. Tracings represent the average of 256 responses. Example is from 8 kHz stimuli. (**D**) Schematic for where cochlear whole mount sections for apical, middle, and basal turns were obtained. Counts for inner (**E**) and outer (**F**) hair cells for each turn show near-total inner and sub-total outer hair cell ablation, respectively, by 4 dpi. (**G**) Counted hair cells have a DAPI-stained nucleus associated with an anti-Myosin 7a-stained cell body. While some damaged inner hair cells are still counted based on these parameters, they may have a swollen and unhealthy morphology.

We used *B6.Cg-Pou4f3tm1.1(HBEGF)Jsto/RubelJ* mice (The Jackson Laboratory, stock #028673), which expressed the human diphtheria toxin receptor (DTR) behind *Pou4f3*, a transcription factor expressed specifically by hair cells in the inner ear^17^. Since mice are resistant to diphtheria toxin (DT), expression of the human DTR gene in hair cells allowed for selective hair cell ablation upon systemic DT administration. DTR transgenic animals experienced rapid hearing loss after DT injection while wild-type (WT) littermates did not. Hearing was assessed by auditory brainstem response (ABR) thresholds (Fig. 1c), which were determined for pure tones (5.7–32 kHz). Mature mice (P30) were injected with DT (6.25 ng/g) and tested 2- and 4-days post injection (dpi). Results were the mean thresholds of 8 ears from 4 animals per condition. WT animals that received DT injection had identical ABRs at 4 dpi compared to uninjected WT controls (Fig. 1a). In DTR animals, impairment progressed rapidly with significant threshold elevations by 2 dpi and no animals had ABRs at the highest intensity presented (95 dB SPL) by 4 dpi (Fig. 1a). This dose of DT continued to have no effect on WT animals by 90 dpi (Fig. 1b), which had identical thresholds to age-matched, uninjected WT controls. Differences in thresholds between WT control animals at P30-32 and P120-122 were attributed to age-related hearing loss characteristic of the C57BL/6J background strain^18^.

Physiologic data was supported by histology. Hair cells were counted along representative sections (200 μm along the contour of the tunnel of Corti) of the apex, middle, and base of the cochleae (Fig. 1d). Cells from each turn of 8 ears from 4 animals per condition were counted. Hair cell loss appeared to be uniform along the sensory epithelium. Inner hair cells were lost more quickly than outer hair cells at the current dose (Fig. 1e,f), which may be reflective of strong Pou4f3 expression in inner hair cells compared to outer hair cells^17^. By 2 dpi, DTR animals had a decreased number of inner hair cells while some remaining inner hair cells had a swollen and unhealthy morphology (Fig. 1g). However, there was no apparent loss of any outer hair cells. By 4 dpi, nearly all inner hair cells were gone while the number of outer hair cells was significantly reduced.

### Vestibular, retinal, and central exclusion

While ablation of cochlear hair cells induced hearing loss, it was important to spare vestibular hair cells so that the effect of hearing loss on spatial learning and memory would not be confounded by vestibular deficits. As previously demonstrated, the ablation of vestibular hair cells required a much higher dose of DT than cochlear hair cells in DTR animals^17,19^. A small dose of DT (6.25 ng/g) caused rapid cochlear hair cell loss but did not cause vestibulomotor dysfunction phenotypes such as head-bobbing, staggering gait, circling or rolling behavior at any point after DT injection.

**Figure 2:**
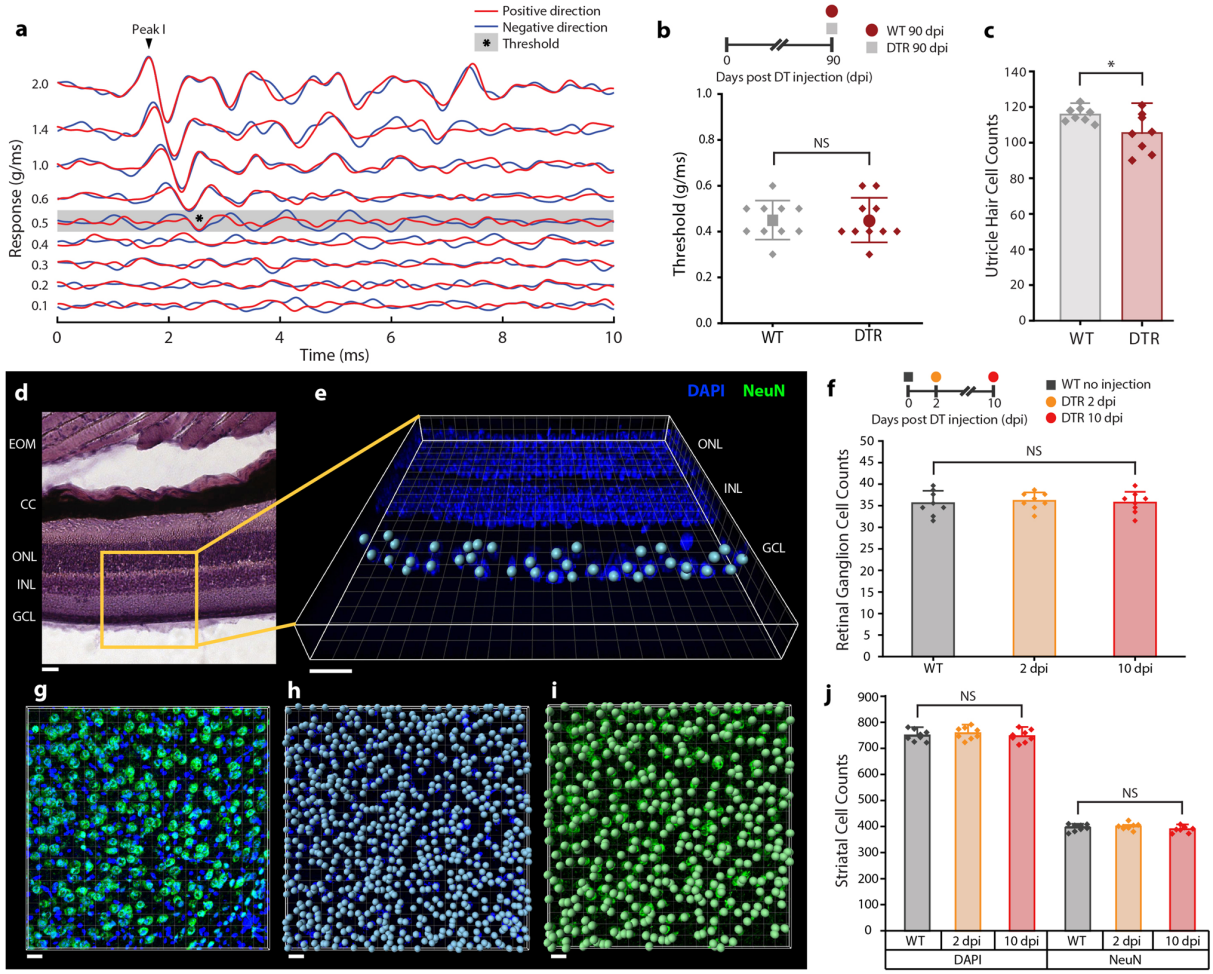
Vestibular, retinal, and central exclusion. Error bars represent standard deviation, *P < 0.05. All scale bars represent 20 μm. (**A**) Vestibular thresholds were determined to be the lowest intensity at which a characteristic Peak 1 could be identified in both positive and negative directions. Tracings represent the average of 256 responses. (**B**) Vestibular thresholds at 90 dpi shows no detectable physiologic vestibular difference between WT and DTR animals (0 dpi = P30, n = 10 mice per group). (**C**) Utricle cell counts per 130 μm^2^ section show a statistically significant reduction in DTR animals that were not physiologically significant. Each point represents the average of a striolar and extrastriolar count per animal (n = 10 mice per group). (**D**) H&E stained retina shows characteristic architecture: extraocular muscles (EOM), choroid coat (CC), outer nuclear layer (ONL), inner nuclear layer (INL), and ganglion cell layer (GCL). (**E**) GCL cell counts were performed in an automated fashion using spot detection in the DAPI channel along 210 μm sections (40 μm original slice thickness). (**F**) No loss of RCG cells were observed at 2 and 10 dpi. Each point represents the average of 6 sections per eye (n = 8 eyes from 4 animals per group). (**G**) Striatal cells were counted in 420  μm^2^ sections (40 μm original slice thickness) using anti-NeuN with DAPI counter-stain. (**H**) Striatal nuclei (all cell types) counts were performed in an automated fashion using spot detection in the DAPI channel. (**I**) Striatal neuron counts were performed in an automated fashion using spot detection in the NeuN channel. (**J**) No loss of striatal nuclei or neurons were detected were observed at 2 and 10 dpi. Each point represents the average of 8 sections per hemisphere (n = 8 hemisphere from 4 animals per group).

Long term vestibular function was objectively assessed using VsEPs (Fig. 2a), histology, and running speed. Results were obtained from 10 animals per condition. WT animals that received DT injection had identical VsEP thresholds compared to DTR animals at 90 dpi (Fig. 2b). Utricle bundle counts at 90 dpi revealed that there was a significant difference between WT and DTR (one-tailed t-test: 115.75 [95% CI 112.18, 119.31] vs 105.37 [95% CI 96.02, 114.73] hair cells, p = 0.028, Fig. 2c). However, the mean utricle hair cell loss of 8.96% was less than the > 60% loss necessary to be physiologically detected^20,21^. Furthermore, when comparing movement metrics between WT and DTR, there was no significant difference in mean movement velocity (one-tailed t-test: 5.766 cm/s [95% CI 5.512, 6.020] vs 5.848 cm/s [95% CI 5.648, 6.048], p = 0.610) and subjective assessment of gait on visual and center-point video tracking analysis revealed no apparent differences between groups.

In the eye, *Pou4f3* is expressed in small subset of retinal ganglion cells (RGCs) and transgenic deletion of *Pou4f3* caused hearing and vestibular defects but no retinal defects^22–25^. It has been previously suggested that DT would not be lethal to RGCs in mature DTR animals^26^. Further considering that the dose response allowed for vestibular sparing despite *Pou4f3* expression in 100% of vestibular hair cells, we hypothesized that the current dose would spare RGCs. To experimentally confirm retinal exclusion, mature mice (P30) were injected with 6.25 ng/g DT and retinas were harvested at 2 and 10 dpi. RGC counts were obtained in an automated fashion along a 210 μm section of the RGC layer (40 μm original slice thickness; Fig. 2d,e) and the average of 6 evenly spaced sections was computed per eye. Results reported were from 8 eyes from 4 animals per condition. There was no difference in RGC counts between WT and DTR animals at 2 and 10 dpi (Fig. 2f; one-way ANOVA: F [2, 21]  = 0.09, p = 0.9133). All animals reacted to visual confrontation and demonstrated seeing behavior throughout the experiment.

While *Pou4f3* mRNA expression has been reported centrally in the striatum and olivary nuclei^17^, there are no prior reports of the gene product (Brn3c) expressed centrally in mature animals to our knowledge. To experimentally confirm central exclusion, mature mice (P30) were injected with 6.25 ng/g DT and brains were harvested at 2 and 10 dpi. Striatal cells were counted in an automated fashion in 420 μm^2^ sections (40 μm original slice thickness; Fig. 2h,i). DAPI was used to label nuclei of all cell types including neurons and glia, while anti-NeuN labeled perinuclear cytoplasm of neurons specifically (Fig. 2g) and the average of 8 equally spaced, atlas-matched sections was computed per hemisphere. Results reported were from 8 hemispheres from 4 animals per condition. There was no difference between WT and DTR animals at 2 and 10 dpi in number of striatal nuclei (one-way ANOVA: F [2, 21] = 0.50, p = 0.6154) or neurons (one-way ANOVA: F [2, 21]  = 1.47, p = 0.2522; Fig. 2j).

### Cochlear hair cell ablation caused a spatial learning/memory deficit

**Figure 3:**
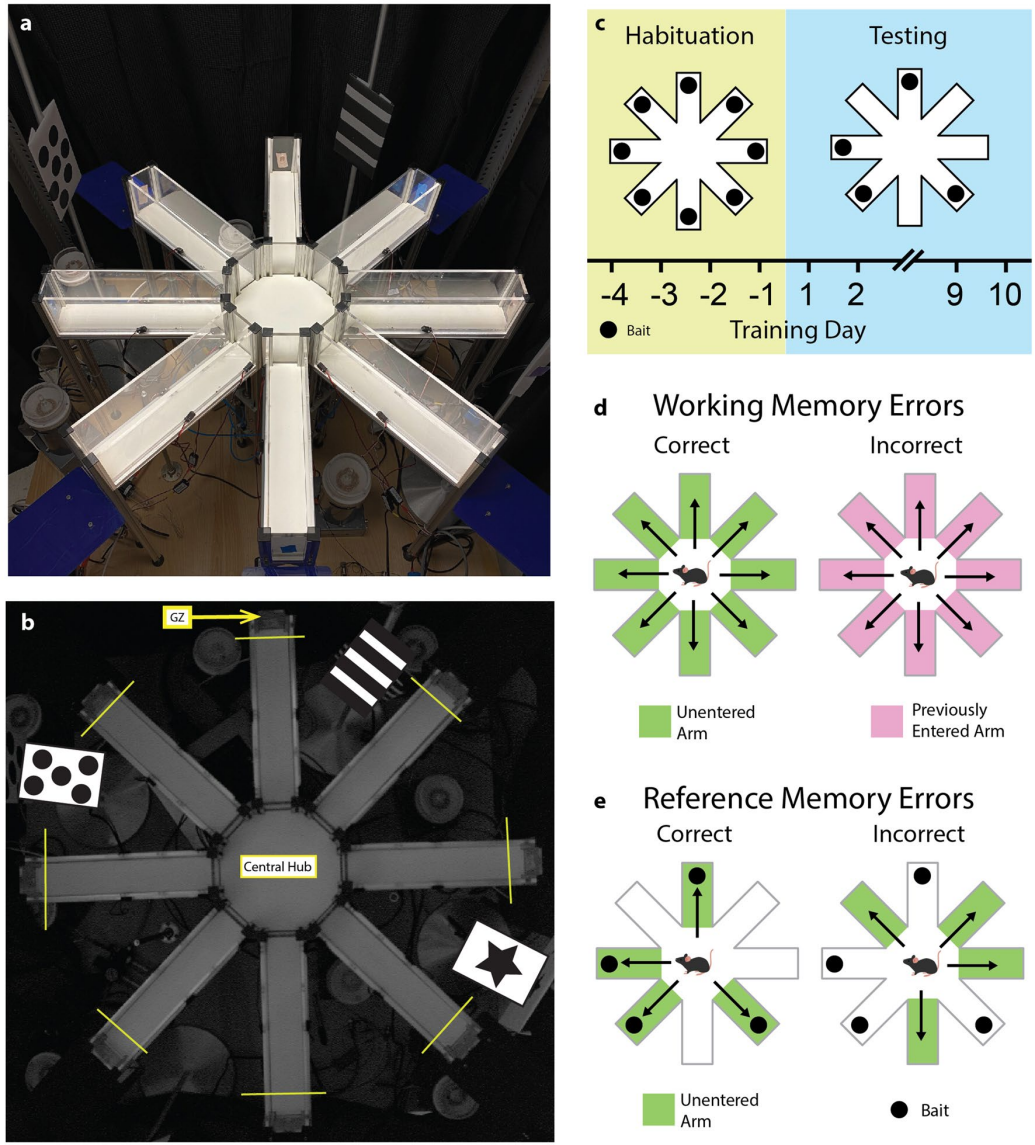
Radial arm maze (RAM) methodology. (**A**) RAM (highlighted) within the testing stage. The RAM is elevated from stage floor and surrounded by curtains. Permanent visual cues are shown in relation to the RAM. (**B**) Image taken from the overhead video tracking system camera shows goal zone (GZ) boundaries for automated video scoring. Presence of a nose-point in a GZ was scored as an “entry”. Configuration of visual cues are shown by an overlaid schematic. (**C**) RAM training timeline consists for 4 days of “habituation” in a fully-baited RAM immediately followed by 10 days of “testing” in a partially-baited maze, where baited arms remained constant throughout the testing period. No training day was assigned “day 0”. (**D**) For working memory, correct entries were first entries into any previously un-entered arm during a trial, while errors were counted each re-entry into a previously visited arm. (**E**) For reference memory, correct entries were first entries into baited arms, while errors were counted for each first entry into an un-baited arm (maximum of 4 reference memory errors per trial).

Spatial learning/memory was assayed using a fully automated 8-arm radial maze and objectively scored using video tracking software (Fig. 3a,b). Food-deprived mice were given 4 daily habituation trials in the radial arm maze (RAM) where mice learned that a single bait exists at the terminal end of 8/8 RAM arms followed immediately by 10 testing days where a single bait was placed at the end of same 4/8 RAM arms each trial (Fig. 3c). The maze was scored in two ways. First, working memory errors (WMEs) were defined as any re-entry into a previously entered arm (Fig. 3d). Working memory informed mice which arms had been previously visited in a given trial. WMEs were independent of baiting pattern and could be scored during habituation and testing. Second, reference memory errors (RMEs) were first entries into never-baited arms (Fig. 3e). Reference memory informed mice which arms would be baited. RMEs relied on the 4/8 RAM configuration and had a maximum score of 4 per trial. Due to the nonparametric nature of the data, group performance on single days were compared using Kruskal–Wallis tests and longitudinal performance was compared using the nonparametric analysis of longitudinal data (nparLD) package for R^27^.

The first experiment determined whether cochlear hair cell ablation caused a measurable difference in spatial learning/memory. DTR and WT animals were injected during P30-P60 and RAM training began during P90-P120. For working memory, DTR animals performed worse during habituation longitudinally (nparLD: F[2.51,28.95] = 12.070, p = 5.93 × 10^–5^). On the last day of habituation (training day − 1), the difference in group performance approached statistical significance (Kruskal–Wallis: χ^2^ = 12.489, p = 0.0059). When the RAM paradigm was changed from habituation to testing, WMEs increased in all groups as expected. However, the difference in WMEs between groups at the beginning of the testing period on day 1 was not significantly different (Kruskal–Wallis: χ^2^ = 2.573, p = 0.4622). When longitudinal performance was statistically compared during the testing period, DTR animals had significantly worse performance than WT animals (nparLD: F[1,35.75] = 35.94, p = 7.23 × 10^–7^).

For reference memory, DTR animals performed worse than WT animals longitudinally during the testing period (nparLD: F[2.47,25.41] = 11.27, p = 0.0001). By the end of the testing period on training day 10, DTR animals committed more errors than WT animals (Kruskal–Wallis test: χ^2^ = 7.985, p = 0.0463). These data demonstrate that lesions isolated to the auditory periphery cause impairment of central processes that do not directly rely on auditory sensory input.

### Effects of hearing loss duration

In order to determine whether the duration of hearing loss had an effect on spatial learning/memory, DTR animals (P60-DT) were divided into two groups based on age at RAM training start (P90 and P120), which represented different durations of hearing loss (30 dpi and 60 dpi, respectively). WT animals were also divided into groups that were age-matched with the DTR groups with respect to age at RAM training start (P90 and P120).

**Figure 4:**
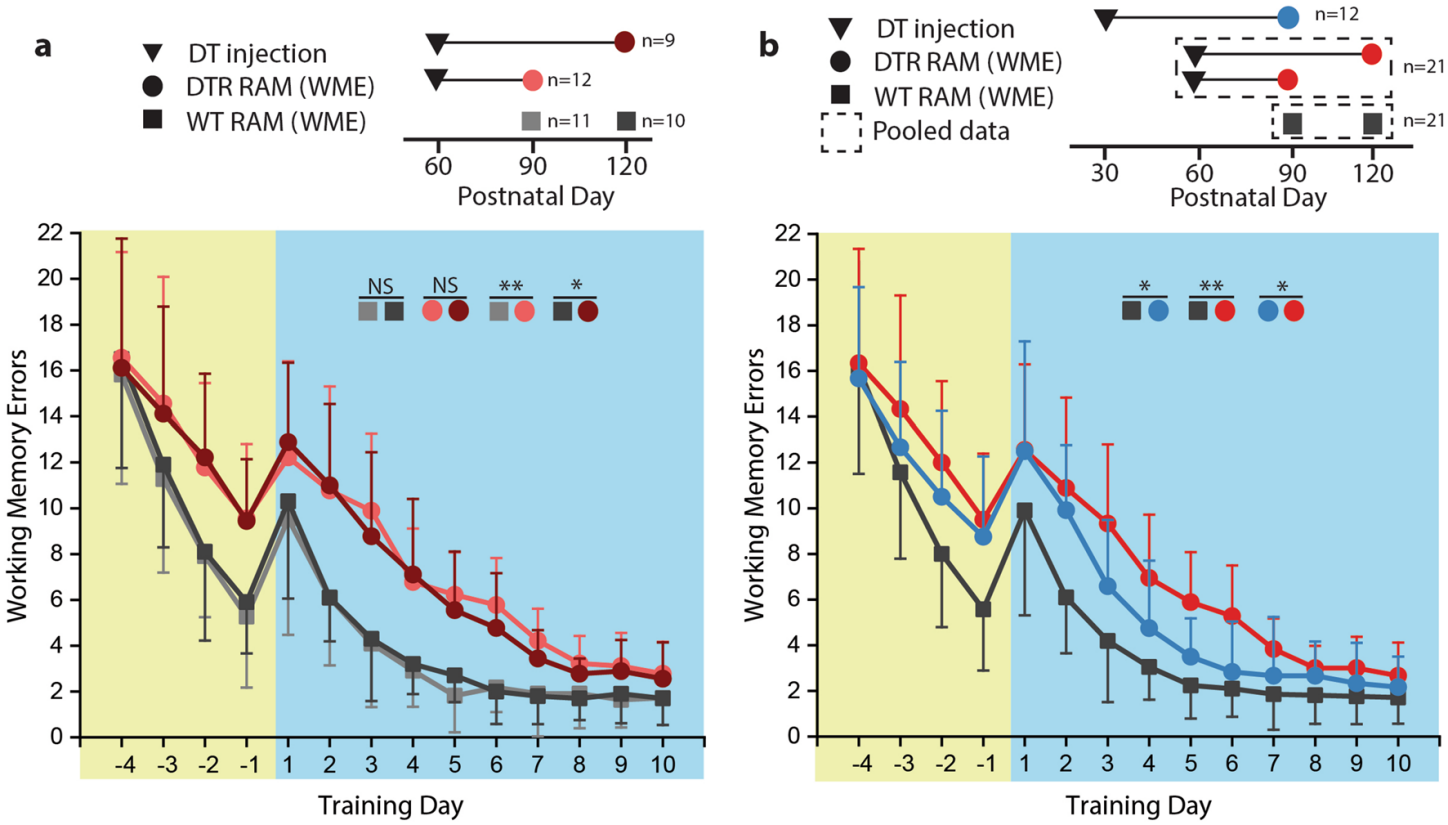
Longitudinal performance for working memory. Error bars represent standard deviation, *P < 0.05, **P < 0.001. Training day 1 corresponds to the indicated postnatal day. (**A**) Longitudinal comparison of working memory errors (WMEs) between DTR mice injected at P60 and WT control animals demonstrate a significantly greater number of WMEs in DTR animals. Duration of hearing loss (DTR animals) and postnatal day (all animals) at training day 1 had no effect on performance. (**B**) Longitudinal comparison of WMEs between DTR animals injected at P30 and P60. WT control animals had fewer WMEs than both DTR groups. However, the DTR group injected at P30 had significantly fewer WMEs than the DTR group injected at P60.

For working memory (Fig. 4a), there was no difference in longitudinal performance between WT groups (nparLD: F[2.36,44.88] = 0.281, p = 0.791), demonstrating that there was no difference in baseline performance by age between P90 and P120. DTR groups performed worse than their WT counterparts (nparLD: F[2.09,37.62] = 5.424, p = 0.008 for P90 and F[3.59,61.07] = 2.708, p = 0.044 for P120). Between DTR groups, there was no difference in performance (nparLD: F[2.75,44.01] = 0.446, p = 0.705), suggesting that duration of hearing loss had no effect on performance.

**Figure 5:**
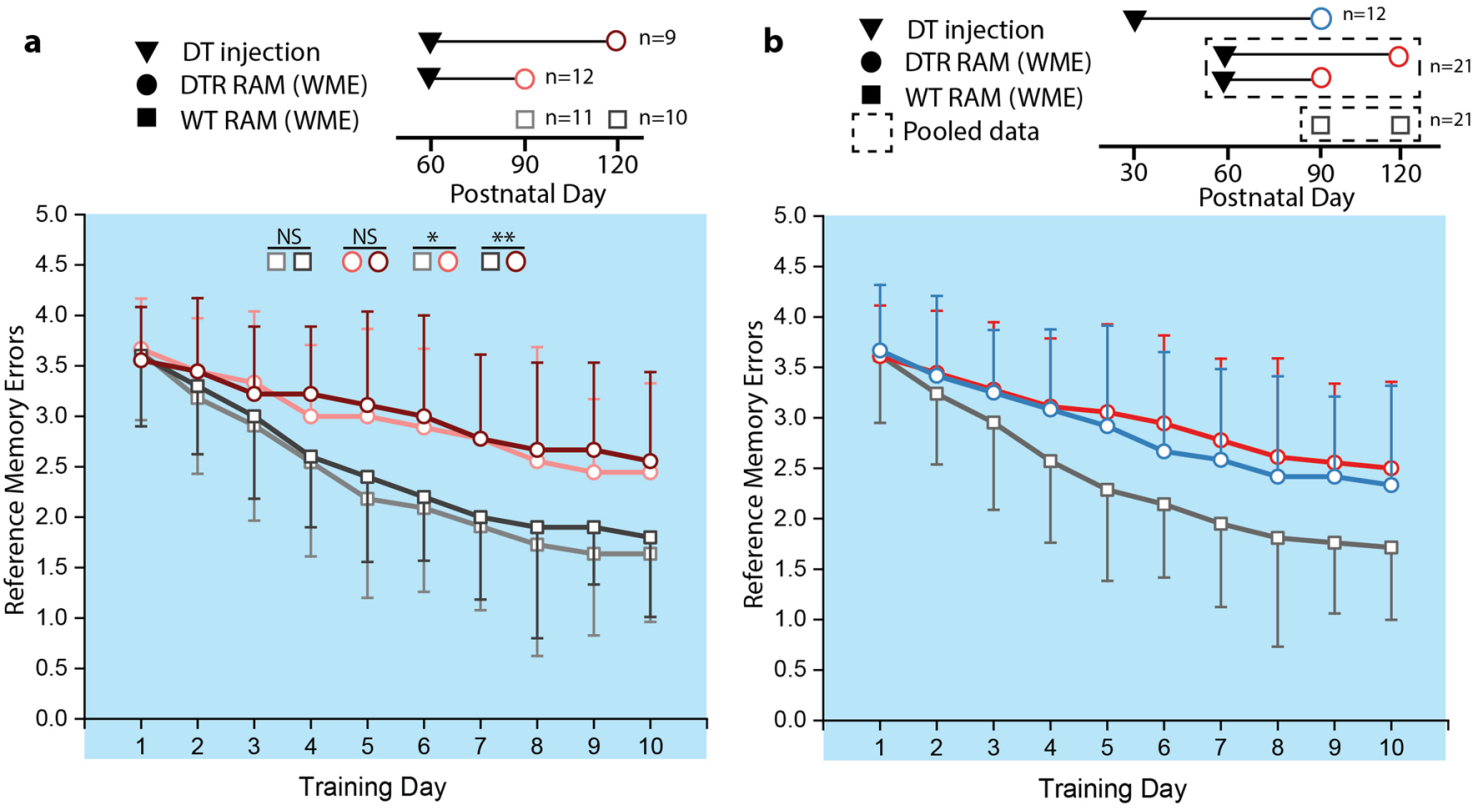
Longitudinal performance for reference memory. Error bars represent standard deviation, *P < 0.05, **P < 0.001. Training day 1 corresponds to the indicated postnatal day. (**A**) Longitudinal comparison of reference memory errors (RMEs) between DTR mice injected at P60 and TW control animals demonstrate a significantly greater number of RMEs in DTR animals. Duration of hearing loss (DTR animals) and postnatal day (all animals) at training day 1 had no effect on performance. (**B**) Longitudinal comparison of RMEs between DTR animals injected at P30 and P60. WT control animals had fewer RMEs than both DTR groups. There was no difference in RMEs between DTR animals injected at P30 versus P60.

Similar associations were observed for reference memory (Fig. 5a). There was no difference in longitudinal performance between WT groups (nparLD: F[1.00,14.15] = 0.775, p = 0.393). DTR group performed worse than their WT counterparts (nparLD: F[1.00,11.66] = 16.55, p = 0.002 for P90 and F[1,14.18] = 17.04, p = 0.001 for P120]. There was no difference in performance between DTR groups (nparLD: F[1.00,12.82] = 0.240, p = 0.632). These data suggest that while hearing loss causes a spatial learning/memory deficit, the duration of hearing loss does not affect the severity of the cognitive deficit in age groups considered.

### Effects of hearing loss age of onset

To assess whether age at onset of hearing loss affected spatial learning and memory, performance of an additional group of DTR animals that received DT injection at a younger age (P30) and began RAM training at 60 dpi (DTR-P30 DT). Since performance was statistically similar between DTR groups injected at P60 and tested at 30 or 60 dpi for both WME (nparLD: F[2.75,44.01] = 0.446, p = 0.705) and RME (nparLD: F[1.00,12.82] = 0.240, p = 0.632), data from these groups were pooled for analysis (DTR-P60 DT). For the same reason, data from WT groups injected at P30 or P60 and tested at 60 dpi were pooled for analysis (nparLD: F[2.36,44.88] = 0.281, p = 0.791 for WME; and F[1,14.15] = 0.775, p = 0.393 for RME).

For working memory (Fig. 4b), longitudinal performance of the DTR-P30 DT group was not significantly worse than the WT group during habituation (nparLD: F[1,26.14] = 3.38, p = 0.0771). However, the DTR-P30 DT group performed significantly worse during testing (nparLD: F[1, 28.34] = 4.75, p = 0.038). The DTR-P60 DT group also performed significantly worse than the WT group during habituation (nparLD: F[2.51,28.95] = 12.07, p = 5.93 × 10^–5^) and during testing (nparLD: F[1,35.75] = 35.94, p = 7.23 × 10^–7^). When comparing the DTR groups, there was no difference in performance during habituation (nparLD: F[1,30.77] = 0.24, p = 0.604), however, the DTR-P60 DT group performed significantly worse than the DTR-P30 DT group during testing (nparLD: F[1,24.46] = 5.34, p = 0.0296), suggesting that age at hearing loss onset may affect working memory.

For reference memory (Fig. 5b), the DTR groups both performed worse than the WT groups over the testing period (nparLD: F[1,26.54] = 18.47 p = 2.06 × 10^–4^ for DTR-P30 DT and F[1,35.32] = 35.39, p = 8.71 × 10^–7^ for DTR-P60 DT). However, there was no difference in performance observed between DTR groups (nparLD: F[1,28.73] = 0.57, p = 0.455). These data suggest that age of acquired hearing loss affects the degree of central deficit and that younger age is associated with attenuated deficit. Since neural plasticity is greater at younger ages, it is likely that increased plasticity compensates for the loss of auditory sensory input.

## Discussion

The results of this study show that isolated sensorineural hearing loss produced via selective cochlear hair cell ablation causes spatial memory impairments. The duration of hearing loss had no impact on the degree of memory impairment in the timelines studied; mice that experienced 1 month of hearing loss demonstrated similar memory deficits to mice that experienced 2 months of hearing loss. However, the age of hearing loss onset affected the degree of memory impairment; mice that lost hearing at P60 had worse memory than mice that lost hearing at P30. These data suggest that the sudden hearing loss compromises cognitive function and this effect may be modulated by the age of hearing loss onset. This finding offers novel insights into the putative pathway by which hearing loss causes cognitive decline in humans.

Rodents rely heavily on auditory information in the form of vocalization for and social integration and establishment of social structures (Brudzynski, 2015). These vocalizations are predominantly ultrasonic (> 20 kHz); while human hearing typically extends from 0.02 to 20 kHz with fine tuning to speech frequencies (0.08 to 2 kHz), rodents hear sounds ranging from 0.2 to 90 kHz^28,29^. Calls at 22 kHz are associated with threats while calls at 50 kHz are associated with rewards^30^. Mouse pups produce isolation vocalizations in the 50–200 kHz range^31^ and reproductive vocalizations in adult mice are recorded at intensities over 100 dB SPL at 40 kHz^32^. Taken together, hearing is an important sensory modality in mice for interpretation of social behaviors, thus making it plausible that acquired hearing loss could have central consequences in mice.

Prior data in mice has demonstrated that the degree of age-related hearing loss characteristic of the C57BL/6 strain closely correlates to the decrease in spatial memory performance and hippocampal plasticity^1,10,11^. This is consistent with human neuroimaging data that demonstrates that age-related hearing loss is associated with decreased brain volume^33–35^. The hippocampus receives direct input from the auditory cortex^36,37^, which facilitates memory formation and consequently guides behavior^38–40^, so it is conceivable that global cognitive function may be affected by auditory deprivation. However, hearing loss experienced in aging is cumulative in nature and its global consequences may be confounded by other effects of age. Studying rapid-onset hearing loss would therefore be informative in this regard. Prior studies where rapid-onset hearing loss was induced relied on noise as a damage model and found that noise-induced hearing loss (NIHL) caused impairments to spatial working and reference memory^12–14^. However, permanent NIHL requires noise exposure of over 120 dB SPL for several hours. This degree of noise exposure may cause profound stress on central pathways, leading to cognitive and emotional consequences that in turn may affect performance on behavioral assays. Distinguishing the effects of the noise exposure itself from subsequent NIHL is challenging for several reasons. First, the adverse impact of noise on learning and memory has consistently been reported previously^40–44^. Second, impulse noise exposure itself causes long term central effects even in the absence of permanent NIHL^16,45^. Therefore, assessing the effects of peripheral sensory loss requires a model where conditional hearing loss could be induced in adulthood with no or minimal central stress.

Here we used a transgenic mouse model that allowed for conditional and selective cochlear hair cell ablation by insertion of the human DTR gene after the Pou4f3 promoter. This promoter is robustly expressed in inner hair cells with lower expression in outer and vestibular hair cells and minimal expression in the central nervous system^17^. Use of a very low dose of DT caused damage that correlated with the degree of Pou4f3 expression; there was near complete inner hair cell damage that caused profound hearing loss, minor utricle hair cell loss that was not physiologically detectable, and negligible central loss. Therefore, the dominant insult in this model was sensorineural hearing loss due to inner hair cell ablation. Here, we find that sudden sensorineural hearing loss causes a spatial memory and learning deficit.

In the current study, age of hearing loss onset affected the severity of the cognitive impairment. The cohort that experienced hearing loss at P60 had more severe working memory impairment than the P30 cohort. Since working memory and neural plasticity both decrease with age, it is possible this modulates the effect that age of hearing loss has on memory. Mice at P30 may have less developed dependency on hearing and consequently a lower barrier to adapt to hearing loss. Alternatively, the P30 brain may be inherently more plastic than the P60 brain and is therefore better able to adapt to sensory loss. It is likely that a combination of these possibilities is at play. Since selective cochlear hair cell ablation in perinatal mice could not be consistently achieved with the current model, future investigation into the effect of congenital versus acquired hearing loss on cognition would be informative. This effect was not observed for reference memory; however, it is likely that this was influenced by the limited resolution of the reference memory assay.

Finally, the duration of hearing loss did not affect the degree of cognitive impairment up to 2 months after onset. It is possible that in sudden hearing loss, the maximal cognitive effect is rapidly reached and persists for the duration of hearing loss. This is consistent with prior data suggesting that partial hearing recovery occurring 12 months after noise-induced hearing loss is accompanied by partial recovery of spatial working memory but not reference memory^14^. Since hearing loss is permanent in cochlear hair cell ablation, it is possible that the cognitive deficit may be alleviated by hearing restoration. Alternatively, it is possible that the current study period falls within the transitional phase following hearing loss, as the mammalian brain is adaptable to other forms of sensory loss. Specifically, visual loss similarly causes cortical and subcortical reorganization that hinders cognitive performance^46^. However, long-term cognitive recovery following perpetual visual loss occurs^47–49^, suggesting that long-term recovery following hearing loss may also occur beyond the observation period.

In conclusion, this work demonstrates that spatial learning and memory impairment follows mature-onset cochlear hair cell ablation in mice, suggesting that sensorineural hearing loss causes a cognitive impairment. It is likely that hearing loss has a similar effect on human cognition and behavior given the strong association observed between hearing loss and cognitive decline. It would be of interest to know whether hearing loss onset before the critical period would have a similar effect on behavior, whether long term recovery occurs, or if this effect could be mitigated by auditory rehabilitation. Additionally, this experimental approach provides the opportunity to investigate the neuroanatomical networks and neurotransmissions involved and influenced by cognitive decline due to hearing loss.

## Methods

### Animals

All experimental procedures were performed on the *B6.Cg-Pou4f3tm1.1(HBEGF)Jsto/RubelJ* transgenic mouse line (DTR; The Jackson Laboratory, stock 028673). The generation and characterization of these mice were previously described^17,19^. In brief, this transgenic line was generated on a C56BL/6 J background and expressed the gene for human diphtheria toxin receptor (*DTR*, *heparin-binding epidermal growth factor receptor*) under the regulation of the *Pou4f3* promoter. The gene product of the *Pou4f3* promoter (Brn3c) is ubiquitously expressed during development but expression in mature animals is limited to cochlear hair cells, vestibular hair cells, and a small subset of retinal ganglion cells (RGCs)^22,50^. While there have been reports of *Pou4f3* mRNA expressed in the striatum and olivary nuclei^17^, there have been no prior reports of the Brn3c expression in the mature brain to our knowledge. Prior work has shown that ablation is dose sensitive^17^, and that low expression of Pou4f3 in off-target tissues such as RCGs allow for off-target exclusion^26^. Additionally, the lack of central Brn3c expression in mature animals would suggest that the central nervous system is also spared in this model.

Systemic treatment of adult, heterozygous DTR mice with a low dose of diphtheria toxin (DT) resulted in specific ablation of cells with high *Pou4f3* expression (i.e. cochlear hair cells) while having no effects on wildtype (WT) littermates. Ablation of vestibular hair cells in DTR mice required a much higher dose of DT than cochlear hair cells^17,19^, thus a single intraperitoneal injection of low dose (6.25 ng/g) DT (Sigma-Aldrich, catalog 322326) at P30 or P60 was chosen to cause hearing loss without apparent vestibulomotor dysfunction. The low dose was then experimentally confirmed to spare RGC and striatal cells. Genotypes were established using PCR as described previously^51^. Animals from both sexes were used. All procedures were approved by the Animal Care and Use Committee at Stanford University. All methods were carried out in accordance with relevant guidelines and regulations.

### Auditory brainstem responses

Auditory brainstem responses (ABRs) were measured as previously described^52^. In brief, mice were anesthetized with an intraperitoneal injection of 10 mg/kg ketamine and 10 mg/kg xylazine and placed on a thermostatic heating pad to maintain body temperature between 37.5–38 °C. ABRs were recorded from a needle electrode placed inferior to the tympanic bulla and referenced to an electrode on the cranial vertex, with a ground electrode placed into the ipsilateral hindlimb. Tone pip stimuli (5 ms duration, 0.2 ms gate time) were presented from an open-field speaker in 10 decibel sound pressure level (dB SPL) steps up from 0 to 90 dB SPL in frequencies ranging from 5.7 to 32 kHz. Waveforms for each condition were generated by averaging 256 responses. ABR thresholds for each frequency were defined as the intensity at which the typical peak I could be identified. A lack of response was designated at the highest sound level, 90 dB SPL.

### Vestibular evoked potentials

Linear vestibular evoked potentials (VsEP) responses were recorded as previously described^53^. Mice were anesthetized (10 mg/kg ketamine and 10 mg/kg xylazine) and had electrodes placed in an identical manner as described for ABRs. The head was clipped to a mechanical shaker, which delivered linear jerk stimuli in the naso-occipital axis ranging from 0.125 to 2.0 g/ms. Broadband noise from an open-field speaker (90 dB SPL) was used to mask responses from the auditory components of cranial nerve VIII. Responses from normal and inverted vestibular stimulus polarities were summed for 256 sweeps per waveform. VsEP thresholds were defined as the intensity at which a typical positive response peak I could be identified.

### Tissue preparation

Mice were anesthetized as previously described and were then perfused via the intracardiac route with 25 mL of 4% paraformaldehyde (PFA). Cochleae, eyes, and brains were then immediately dissected. Cochleae were perfused with 0.5 mL of PFA via the intralabyrinthine route through openings made in the round and oval windows, immersed in PFA for 45 min, decalcified in 1 M EDTA for 3 days at 4 °C, and finally dissected into 3 turns^54^. Turns were triple-washed in PBS with 0.5% triton (PBST) then incubated for 1 h in blocking solution (4% bovine serum albumin in PBST), overnight at 4 °C in rabbit anti-myosin 7a (1:500 in PBST; Proteus Bioscience; catalog 25–6790), then for 1.5 h in Alexa Fluor donkey anti-rabbit 546 (1:600; Invitrogen, catalog A10040) and DAPI (1:10,000; Invitrogen, catalog D1306).

Eyes were immersed in PFA for 15 min, then 30% sucrose in PBS overnight at 4 °C, embedded in optimal cutting temperature (OCT) compound (VWR International, catalog 4583), then cryosectioned along the transverse axis in 40 μm-thick sections onto slides. Slides were then stained with hematoxylin and eosin (H&E; Fisher Scientific, catalog 9990001) or DAPI.

Brains were immersed in PFA overnight, then 30% sucrose in PBS for 48 h at 4 °C, embedded in OCT, then cryosectioned along the antero-posterior axis in 40 μm-thick sections into wells of PBS. Free floating sections were then triple-washed in PBST, incubated for 1 h in blocking solution, 2 h in rabbit anti-NeuN (Invitrogen, catalog PA578639), then 1.5 h in Alexa Fluor donkey anti-rabbit 546 and DAPI.

All tissues were imaged as Z-stacks on a Zeiss LSM700 confocal microscope. Images were captured using Zen Black software (Zeiss) and analyzed with Zen Blue software (Zeiss) and ImageJ (NIH) or Imaris (Oxford Instruments).

### Cell counting

Cochlear hair cells were counted along 200 μm sections along the contour of the tunnel of Corti. Cells with DAPI-stained nuclei associated with myosin 7a-stained cell bodies were counted. Counts were statistically compared between groups using one-way ANOVAs. Vestibular hair cells were counted in 130 μm × 130 μm sections of the utricle. Cells with Phalloidin-stained bundles were counted in a striolar and an extra-striolar section and averaged for each utricle. Counts were statistically compared using t-tests.

For RGCs, nuclei in the RGC layer were counted along 210 μm sections along the contour of the inner limiting membrane. Counts were performed in an automated fashion using spot detection in Imaris. The automatic threshold at which spots were inserted in the RGC layer was manually adjusted on a representative image, the parameters saved, then batch applied across all retinal sections for objective counts relative to the reference image. For each eye, the RGC count was the average of six evenly spaced sections along the transverse axis. Counts were statistically compared between groups using one-way ANOVAs.

Striatal cells were counted in 420 μm × 420 μm sections. Counts were performed in an automated fashion using spot detection in Imaris. Automatic thresholds at which spots were inserted in the field of view were manually adjusted on a representative image separately for NeuN and DAPI channels, the parameters saved, then batch applied across all striatal sections for objective counts relative to the reference image for each channel. For each hemisphere, the striatal cell count was the average of eight equally spaced, atlas matched (Allen Mouse Brain Atlas) sections across the antero-posterior axis. Counts were statistically compared between groups using one-way ANOVAs.

### Behavioral experiment

Animals were housed in standard tub cages in groups of 3–4 littermates of identical sex, genotype, and DT injection status prior to experiments. All cages were enriched with corn cob bedding, paper nesting material, and paper tubes equal in number to the animals in each cage. Animals were allowed access to regular feed and water ad libitum and maintained on a 12:12 light cycle (6 am to 6 pm light). Animals were transitioned to individual housing and feed consisted of pellets of identical size and weight (20 mg/pellet; Bio-Serve, catalog F0163) approximately one week prior to experiments. During this time, animals were reduced to 85%-90% of their free-feeding weight through feed restriction and acclimated to transport in their home cages and to experimenter handling via a non-aversive tunnel handling technique^55,56^. All procedures were performed during the light cycle.

Spatial learning/memory was assayed using an automated 8 arm radial maze (Fig. 3a; MazeEngineers, Cambridge, MA, USA) integrated with EthoVision XT video tracking software (Noldus, Leesburg, VA, USA). The radial arm maze (RAM) consisted of an octagonal central hub (40 cm perimeter) and eight arms (35 cm length, 5 cm width, 10 cm wall height each). An overhead camera was mounted 165 cm above the maze floor. The maze floor was white to maximize video contrast with the animals’ black coats, while the walls, doors, and lids were transparent that allowed animals to visualize extra-maze cues (Fig. 3b), which remained constant throughout the experiment. Guillotine-style doors were powered by a pneumatic system underneath the maze. The maze was placed on a square stage, the maze floor was elevated 50 cm above the stage floor and surrounded by opaque black curtains on all sides extending 215 cm above the stage floor. Diffuse light sources within the apparatus maintained illuminance of 30 lx at the central hub. The entire apparatus was placed in a room exclusively used for RAM testing.

At the beginning of each trial, mice were placed in a RAM and restricted to the central hub to promote spatial orientation. Once all 8 octants of the central hub were explored and 30 s had elapsed, all doors opened to allow free exploration of the maze. During habituation, single feed pellets served as bait were placed in goal zones located at the terminal ends of each arm (Fig. 3b). After returning to the central hub, animals were restricted there for 3 s in order to prevent serial search strategy, whereby adjacent arms are visited in a clockwise or counterclockwise fashion. In order to minimize visual, auditory, and tactile startle caused by simultaneous guillotine door closure, doors for all arms except the occupied arm were closed upon nose poke into a goal zone, and the final door was closed upon re-entry of the tail base into the central hub.

RAM training occurred over 14 continuous days and consisted of habituation (4 days) and testing (10 days) phases (Fig. 3c). During habituation, all arms were baited (8/8 RAM) and animals learned that a single bait existed in the goal of zones of each arm. Animals that took all baits within a 10-min trial by the last day of habituation were advanced to the testing phase. During testing, 4/8 arms were baited for each trial (4/8 RAM). The always-baited and never-baited arms were kept constant throughout the testing phase.

Scoring was performed in an automated fashion using video tracking software. An entry was defined as the first nose poke into a goal zone for a given arm entry (Fig. 3b). Working memory errors (WME) were scored for all re-entries into a previously entered arm (Fig. 3d). WMEs were scored during habituation and testing because it could be scored independent of the baiting pattern. Reference memory errors (RME) were scored for all first-time entries into never-baited arms (Fig. 3e). RMEs were scored only during the testing phase because it required the 4/8 baiting pattern and had a maximum score of 4 for each trial. An example video for scoring is shown in Supplemental Materials (Video S1).

### Statistical analyses

Statistical analyses were performed using R software (R Foundation for Statistical Computing, Vienna, Austria). Measures of auditory damage were compared between groups using two-way ANOVAs or t-tests. Since behavioral data was nonparametric due to scoring ceilings and floors, group performance on specific days were compared using Kruskal–Wallis tests and while longitudinal performance between groups were compared using compared using the nparLD (non-parametric analysis of longitudinal data) package^27^. Results for nparLD analyses were reported as modified ANOVA-type statistics accounting for whole-plot factors.

## Supplementary information

Supplementary Video 1.

Supplementary Information 1.
