# Supplementary material for: Effects of cochlear hair cell ablation on spatial learning/memory
Source: Sci Rep. 2020 Nov 26;10:20687. doi: 10.1038/s41598-020-77803-7 (PMC7692547; doi:10.1038/s41598-020-77803-7)
Supplement: Supplementary file 2 — Supplementary Information 1. [file 41598_2020_77803_MOESM2_ESM.docx]

**Legend for the Video**

*Title:*

Effects of Cochlear Hair Cell Ablation on Spatial Learning/Memory

*Authors:*

Z. Jason Qian and Anthony J. Ricci

*Affiliations:*

Department of Otolaryngology-Head and Neck Surgery, Stanford University School of Medicine

*Legend for the Video:*

Example of automated video scoring for working memory errors (WME) and reference memory errors (RME). WMEs were any re-entry into previously entered arms. RMEs were any first-time entries into never-baited arms. Mouse shown is training day 10.
